# The Adverse Impact of the COVID-19 Pandemic on Abdominal Emergencies: A Retrospective Clinico-Pathological Analysis

**DOI:** 10.3390/jcm10225254

**Published:** 2021-11-11

**Authors:** Elena Vissio, Enrico Costantino Falco, Gitana Scozzari, Antonio Scarmozzino, Do An Andrea Trinh, Mario Morino, Mauro Papotti, Luca Bertero, Paola Cassoni

**Affiliations:** 1 Pathology Unit, Department of Medical Sciences, “Città della Salute e della Scienza di Torino” University Hospital, University of Turin, 10126 Turin, Italy; elena.vissio@unito.it (E.V.); enricocostantino.falco@unito.it (E.C.F.); doanandrea.trinh@unito.it (D.A.A.T.); paola.cassoni@unito.it (P.C.); 2 Hospital Medical Direction, Molinette Hospital, “Città della Salute e della Scienza di Torino” University Hospital, 10126 Turin, Italy; gscozzari@cittadellasalute.to.it (G.S.); ascarmozzino@cittadellasalute.to.it (A.S.); 3 General Surgery 1U, Department of Surgical Sciences, “Città della Salute e della Scienza di Torino” University Hospital, University of Turin, 10126 Turin, Italy; mario.morino@unito.it; 4 Pathology Unit, Department of Oncology, “Città della Salute e della Scienza di Torino” University Hospital, University of Turin, 10126 Turin, Italy; mauro.papotti@unito.it

**Keywords:** COVID-19, surgical emergency, abdominal emergency, emergency department, pathology

## Abstract

The COVID-19 pandemic has caused a worldwide significant drop of admissions to the emergency department (ED). The aim of the study was to retrospectively investigate the pandemic impact on ED admissions, management, and severity of three abdominal emergencies (appendicitis, diverticulitis, and cholecystitis) during the COVID-19 pandemic using 2017–2019 data as a control. The difference in clinical and pathological disease severity was the primary outcome measure while differences in (i) ED admissions, (ii) triage urgency codes, and (iii) surgical rates were the second ones. Overall, ED admissions for the selected conditions decreased by 34.9% during the pandemic (control: 996, 2020: 648) and lower triage urgency codes were assigned for cholecystitis (control: 170/556, 2020: 66/356, *p* < 0.001) and appendicitis (control: 40/178, 2020: 21/157, *p* = 0.031). Less surgical procedures were performed in 2020 (control: 447, 2020: 309), but the surgical rate was stable (47.7% in 2020 vs. 44.8% in 2017–2019). Considering the clinical and pathological assessments, a higher percentage of severe cases was observed in the four pandemic peak months of 2020 (control: 98/192, 2020: 87/109; *p* < 0.001 and control: 105/192, 2020: 87/109; *p* < 0.001). For the first time in this study, pathological findings objectively demonstrated an increased disease severity of the analyzed conditions during the early COVID-19 pandemic.

## 1. Introduction

In Italy, the earliest appearance of COVID-19 was confirmed on the 30 January 2020 [1]. Thereafter, an outbreak of infections started in Lombardy, an Italian Northern Region, and extended progressively to the whole national territory [2]. In Piedmont, a region adjacent to Lombardy, the first case was reported on 22 February and in our hospital, the first COVID-19-positive patient was diagnosed on 5 March. In the following weeks, our institution, the main regional healthcare hub, rapidly admitted an increasing number of COVID-19 patients, reaching the total count of 2486 COVID-19 patients managed up to 31 December 2020.

The COVID-19 pandemic plunged the Italian health system into an extraordinary state of emergency, with many hospitals being dedicated exclusively to COVID-19 patients’ assistance, while other institutions, including our tertiary-level hospital, were forced to rapidly change their workflow to admit both positive and negative patients through a fast and massive reorganization of wards and services. This reorganization also reshaped emergency care, as well as patients’ behaviors and attitudes towards healthcare services. During the first weeks of the COVID-19 pandemic, a strong reduction in emergency department (ED) visits was reported in many countries [3,4,5,6,7,8,9,10,11,12,13,14,15] due to patients’ fear of becoming infected. This delay in seeking medical attention could have negatively affected patients’ management and outcomes, especially when dealing with life-threating or oncological conditions [16,17,18,19], but also with other time-dependent medical emergencies/urgencies [20].

In our hospital, we registered an overall strong reduction of ED admittances during the COVID-19 pandemic and, among the ED-relevant illnesses, abdominal surgical emergencies represent an important subset in which delayed surgical intervention can quickly translate into unfavorable outcomes. Moreover, these kinds of urgent conditions often lead to surgical resection of the involved organ, enabling an assessment of the pathological findings to investigate whether and to what extent COVID-19-induced delays have really increased disease severity. Previous retrospective observational studies [21,22,23] reported a reduction of ED admissions for abdominal surgical emergencies during the COVID-19 infection waves, also suggesting an increase of disease severity at presentation based on clinical [24] and radiological [21,22,23,24,25] data, but an analysis and correlation with pathological findings is still missing.

The aim of the present study was to investigate the association between the COVID-19 pandemic throughout 2020 and ED presentation patterns and management of abdominal surgical emergencies, including an analysis of the pathological findings of surgical specimens. Specifically, data concerning three main abdominal conditions (i.e., diverticulitis, cholecystitis, and appendicitis) were analyzed.

## 2. Materials and Methods

### 2.1. Study Setting

The present retrospective study was conducted at the Molinette Hospital within the “Città della Salute e della Scienza” university hospital, a tertiary hub sited in Turin, the capital city of the Piedmont Region, a Northern Italy region with approximately 4 million inhabitants. Molinette Hospital provides services concerning all medical and surgical specialties, except for the pediatric, obstetrical-gynecological, and orthopaedical-traumatic units, which are hosted in different hospitals within the same hub. Molinette Hospital counts approximately 1200 beds, and its ED admits about 70,000 patients annually. The hospital hosts two pathology units, managing approximately 25,000 surgical specimens annually.

To understand the specific pandemic evolution and context in our country, region, and hospital during 2020, it should be noted that on the 23 February 2020, a first national decree banned public activities and gatherings and blocked people’s movement across different cities/towns. Initially, these restrictions were limited to the Lombardy’s most involved territories. However, in March 2020, a full lockdown was progressively extended to the whole national territory. These limitations remained practically unchanged until the 4 May 2020 when they were progressively reduced until October 2020 when, due to the raising rate of infections, they were reintroduced.

### 2.2. Data Collection

For the purpose of the study, we collected retrospective data from triage/clinical charts of patients who accessed the ED due to the present study conditions between 1 March and 31 December 2017–2020 (2020 as the COVID-19 pandemic year and the mean values of the previous three years as a control series). The inclusion criterion was a diagnosis of one of the three analyzed conditions at the time of ED discharge or transfer, while patients under 18 y/o were excluded. If the same patient visited the ED two or more times during the study period, each visit was considered as a different episode. Pathological records within the same timeframe were searched to collect data regarding the surgically resected samples.

The following data were collected: patients’ urgency triage code (classified by the triage nurse as white, i.e., non-urgent condition; green, i.e., minor urgency; yellow, i.e., major urgency; or red, i.e., emergency), patients’ management in terms of surgical procedures, and, for patients undergoing a surgical procedure, the pathological characteristics of the resected specimen.

For patients who underwent a surgical resection, clinical and pathological findings were manually reviewed to assess disease severity and investigate possible variations due to the pandemic. Specifically, we developed a 3 tier clinical severity score based on clinical findings, attributing to clinical group (CG) 0 patients described by the clinician as affected by a chronic condition, to CG1 patients with an acute condition, and to CG2 patients with a suspected perforation of the involved organ. Similarly, we applied a pathological severity score according to the gross and microscopic findings described on the pathological report: in the pathological group (PG) 0, we included specimens with features suggesting a chronic disease (e.g., chronic inflammation, fibrosis); in PG1 samples with an acute involvement (e.g., granulocytic infiltrate); and in PG2 samples with signs of serosal involvement (e.g., transmural inflammation, serositis, plain perforation). Pathological evaluation was based on the assessment of histological reports without a revision of specimens to avoid any study-induced bias. Data were pseudonymously stored in an electronic database (Microsoft Excel 365, version 16, Redmond, WA, USA).

The study was conducted in accordance with The Code of Ethics of the World Medical Association (Declaration of Helsinki) for experiments involving humans and within the guidelines and regulations defined by the Research Ethics Committee of the University of Turin. Considered the retrospective nature of the study and that there was no impact at all on patients’ care, a specific written informed consent was not required.

### 2.3. Primary and Secondary Outcome Measures

The primary outcome measure was the difference in clinical and pathological disease severity between the COVID-19 pandemic months of 2020 and the corresponding time periods of 2017–2019 while differences in terms of (i) ED admissions, (ii) triage urgency codes, and (iii) surgical rates were the secondary outcome measures.

### 2.4. Statistical Analyses

Categorical variables are reported as frequencies and percentages, whereas continuous variables are reported as means due to a normal distribution of data, confirmed by the Shapiro–Wilk normality test. Univariate analysis was performed with the chi-squared test and Fisher’s exact test for categorical data to compare: (i) the rates of severity triage codes between the same months of 2020 and the control years, and (ii) the distribution of clinical and pathological severity groups between the two time periods (March–December 2020 and 2017–2019). Moreover, we performed the same test considering only the 4 peak months (March, April, May, and November). In all tests, the statistical significance level was set at the conventional *p* < 0.05. The results were analyzed using the Stata/SE 15 statistical software (Stata Corp., College Station, TX, USA).

## 3. Results

### 3.1. Analysis of ED Admissions

Between 1 March and 31 December 2020, 648 patients were admitted to our ED due to the three analyzed causes of acute abdomen (appendicitis, diverticulitis, and cholecystitis) compared to 996 mean accesses during the same months of the three previous years (−34.9%). This decrease was detected in the context of a sharp lowering of all ED admission since the pandemic development (Supplementary Material Table S1). A more prominent reduction was observed during March, April, and November with a partial recovery during the summer period. Considering the three conditions separately, a more pronounced decrease was registered for diverticulitis (135 vs. 262, −48.5%) and cholecystitis (356 vs. 556, −36.0%), whereas it was limited for appendicitis (157 vs. 178, −11.9%).

**Figure 1:**
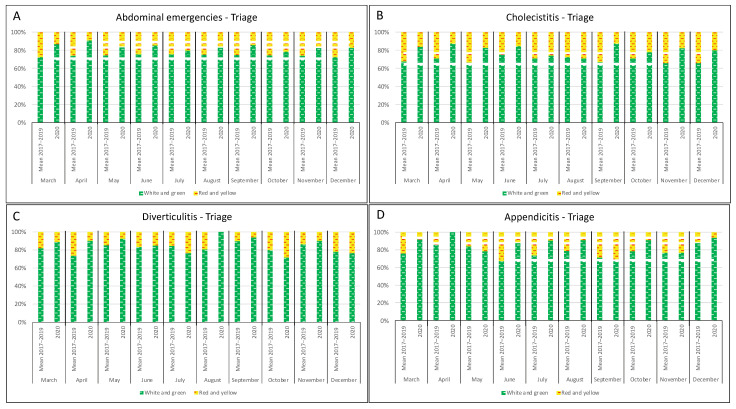
Monthly distribution of patients admitted to the ED for overall abdominal emergencies (**A**), cholecystitis (**B**), diverticulitis (**C**), and appendicitis (**D**) according to the triage classification system: comparison between the 2017–2019 period and 2020.

According to the color-coded triage, less cases received more urgent codes (red and yellow) in 2020 compared to 2017–2019 for cholecystitis (control: 170/556, 2020: 66/356, *p* < 0.001) and appendicitis (control: 40/178, 2020: 21/157, *p* = 0.031) but not for diverticulitis (control: 45/262, 2020: 19/135, *p* = 0.426) (Figure 1, Supplementary Material Table S1).

### 3.2. Analysis of Surgical Procedures

Among the ED-admitted patients, 309 underwent a surgical intervention during the considered time period of 2020, namely 33 for acute diverticular disease, 209 for gallbladder disease, and 67 for appendicular disease and a reduction was noted comparing these data with the 2017–2019 years. In particular, surgical interventions for cholecystitis (209 vs. 321.0, −34.9%) showed the highest drop, although surgeries for diverticulitis and appendicitis also decreased (33 vs. 45.3, −27.2% and 67 vs. 80.3, −16.6%, respectively).

Despite this decrease in the absolute numbers of surgical resections, the surgical rate among the ED-admitted patients was similar: 47.7% in 2020 compared with 44.8% of the pre-COVID-19 years 2017–2019.

Considering the distribution of the surgical interventions during the 2020 months and comparing them with the reference period, we noted a sharp drop during March, a slow recovery during the April–June trimester, and a new decrease during the rest of the year, i.e., during the so-called ‘second wave’ of the pandemic, except for a small peak in September (Supplementary Material Figure S2A).

To understand whether the three pathologies shared the same behavior, we considered their monthly distribution separately. In 2020, cholecystectomies mirrored the general trend (Supplementary Material Figure S2B), while appendicectomies did not show a clear drop neither in March nor during the last 2020 months and surgical procedures due to diverticulitis showed a clear decrease in March only (Supplementary Material Figure S2C,D). Nevertheless, interpretation of the appendicitis and diverticulitis-related surgical procedures is hampered by the low sample sizes.

### 3.3. Analysis of Clinical and Pathological Severity

**Figure 2:**
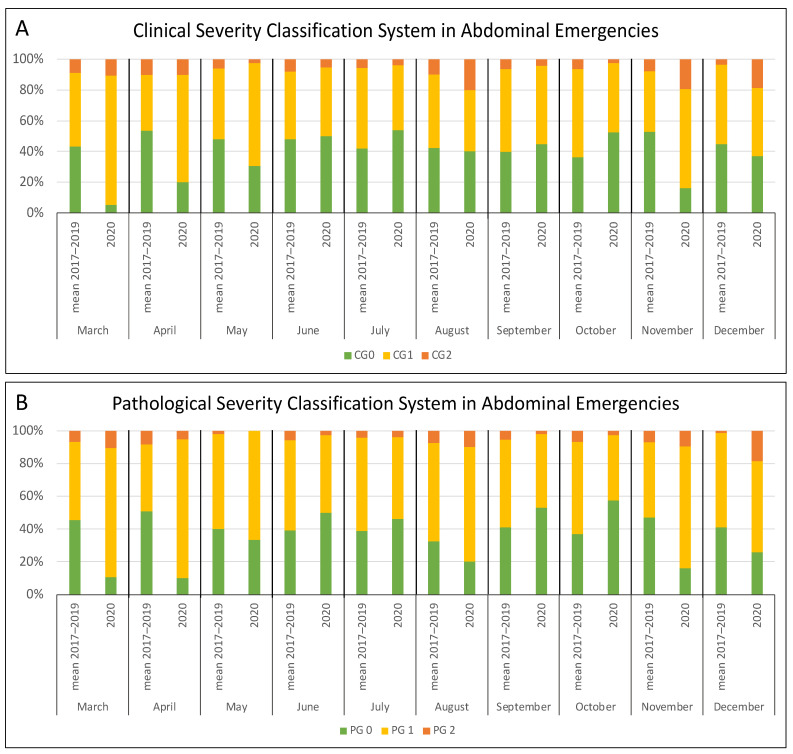
Monthly distribution of patients undergoing surgery for abdominal emergencies according to the clinical (**A**) and pathological (**B**) severity classification system: comparison between the 2017–2019 period and 2020.

To investigate possible changes in the severity of abdominal diseases during the pandemic months, we stratified patients according to their clinical presentation, applying a clinical severity classification scoring system (see the Methods section) to the surgical population of the analyzed 4 years (2017–2020). Analyzing the abdominal emergencies altogether, we noted a higher percentage of severe cases (CG1 and CG2) in 2020 (193/309 in 2020 vs. 246/447 in control period; *p* = 0.042). Considering the four months of the pandemic peak in our region (March, April, May, and November), the increase of acute cases was even more pronounced (87/109 vs. 98/192; *p* < 0.001) (Figure 2A).

**Figure 3:**
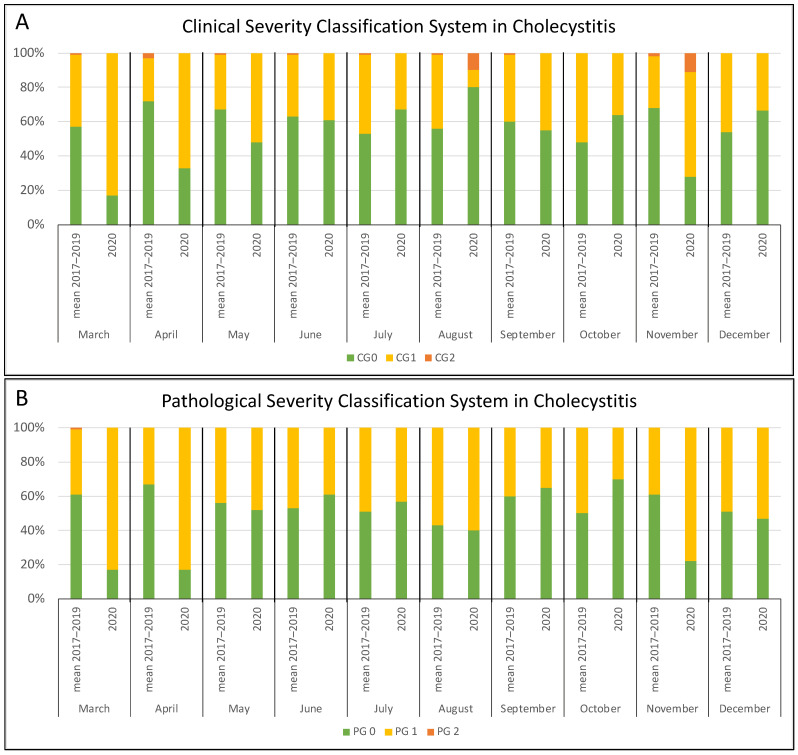
Monthly distribution of patients undergoing surgery for cholecystitis according to the clinical (**A**) and pathological (**B**) severity classification system: comparison between the 2017–2019 period and 2020.

**Figure 4:**
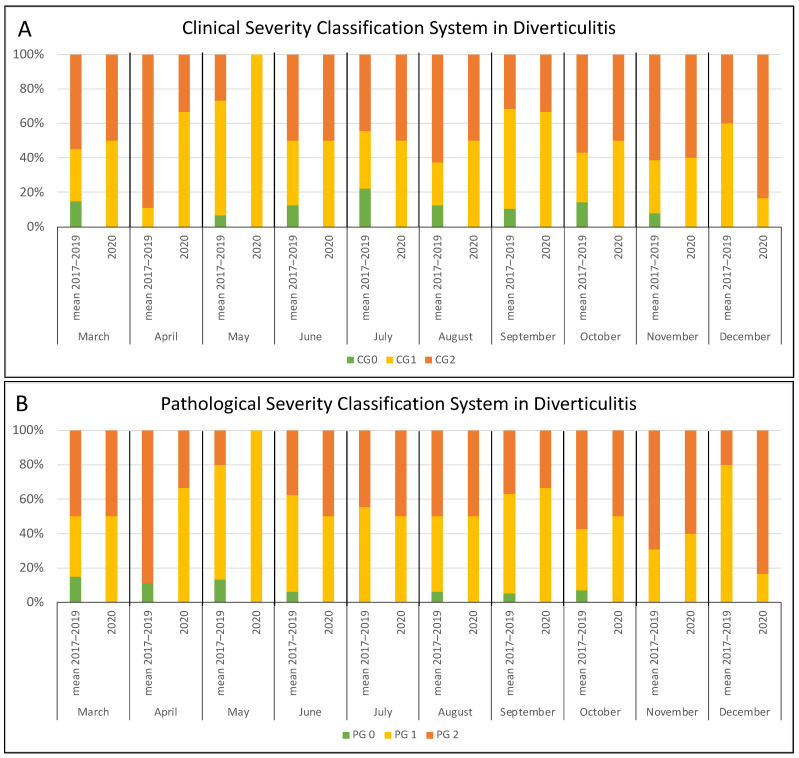
Monthly distribution of patients undergoing surgery for diverticulitis according to the clinical (**A**) and pathological (**B**) severity classification system: comparison between the 2017–2019 period and 2020.

Considering the three conditions separately, clinical severity showed a similar trend, although not statistically significant over the whole year. Restricting the analysis to the four pandemic peak months, the increase of severe cases in 2020 vs. the control period was instead significant for cholecystitis (38/59 vs. 48/138; *p* < 0.001) (Figure 3A) but not for appendicitis (Supplementary Material Figure S3A) and diverticulitis (Figure 4A).

Variations in terms of the CG1/CG2 ratio were variable.

To confirm the clinical results, we further applied a pathological severity classification scoring system to the surgical specimens of the same population. A higher number of acute cases (PG1 and PG2) was observed, but it was not significant when considering the whole year (196/309 vs. 262/447; *p* = 0.180), even if a similar trend was registered. However, considering only the four critical months of 2020, the increase of severe cases was significant (87/109 vs. 105/192; *p* < 0.001) (Figure 2B). The pathological findings showed a trend similar to the clinical assessment by evaluating the conditions separately (Figure 3B and Figure 4B, Supplementary Material Figure S3B), but a significant difference was noted only for cholecystitis in the four peak months (40/59 vs. 54/138; *p* < 0.001).

## 4. Discussion

During the 2020 COVID-19 outbreak, a global reduction in ED visits was described [9,10], both for traumatic and non-traumatic conditions [9] and including conditions requiring urgent attention. For instance, admissions for ischemic strokes almost disappeared in an ED of Piacenza, a small town near Milan [26].

Focusing on abdominal surgical conditions, a pandemic-related reduction has been described for urgent cases [27,28], including appendicitis [21] and diverticulitis [22,24], while no univocal data have been reported for cholecystitis [28].

Our data are consistent with these reports: a decrease of about one-third (−34.9%) of ED admissions due to abdominal surgical emergencies was observed in 2020 and in particular, admissions due to diverticulitis and cholecystitis showed the sharpest declines (−48.5% and −36.0%, respectively). As expected, changes in ED admissions followed the pattern of the COVID-19 pandemic waves in our territory, resulting in higher decreases in March-April (first wave) and November (second wave).

The reduction of global ED presentations has been explained both by fear of becoming infected with COVID-19 in a hospital setting [10,29] and by the effect of lockdown, such as a decrease of traumatic injuries in street and workplace accidents [6,29]; a decrease in cardiovascular accidents due to a reduction of working and pleasure activities; a decrease of respiratory symptoms due to improved air quality [29,30]; and a decrease in respiratory infections due to public health measures, such as school closure, social distancing, and improved hand hygiene [31]. Additionally, the decrease in elective surgical activity might have reduced ED accesses for postoperative complications [5].

Focusing on abdominal surgical conditions, other potential explanations for ED admission reduction are pandemic-related modifications in lifestyle habits, such as changes in eating habits decreasing acute conditions, such as acute cholecystitis [32].

Regarding patients’ management, an increase in conservative treatments has been described, leading to the discharge of the patient, followed by a re-admission after a few days because of a worsening of symptoms [33]. However, according to our experience, the rate of ED-admitted patients who underwent a surgical intervention was similar between 2020 (47.7%) and the previous years (44.8%). This result was made possible by the prompt creation, during the early days of the pandemic, of dedicated workflows within our ED for COVID-19-positive patients with separate triage and examination areas as well as a dedicated operating room. Fast evaluation of nasopharyngeal swabs was also made available 24/7 for ED cases to rapidly assign patients to the right units. Thus, surgical activity was overall preserved, as also shown by our previous report focused on neoplastic conditions [34].

Nevertheless, the most pressing question is if the COVID-19-induced delay in seeking medical care could have led to increased disease severity at the time of ED admittance, thus we investigated this possibility in the present series through multiple approaches.

Firstly, we compared the ED triage codes assigned in 2020 vs. the previous years: urgent codes (red and yellow) decreased in 2020 compared to 2017–2019 for cholecystitis (*p* < 0.001) and appendicitis (*p* = 0.031). This observation could suggest that COVID-19 had no negative consequences, but it collides with two other findings: (i) the surgical rate among admitted patients was similar between 2020 and 2017–2019, and (ii) the analysis of clinical and pathological features of surgical patients/specimens suggested a severity increase for the 2020 months. Although it is difficult to certainly pinpoint the reasons behind this discrepancy, a possibility could be that non-COVID-19-related conditions were perceived as less urgent during the early months of the pandemic both by patients themselves and triage operators due to the ongoing novel quickly evolving health threat.

Secondly, we focused on cases treated by surgical resection to enable a comparison between clinical and pathological findings. Analysis of clinical data found a good agreement between clinical and pathological assessments as well as a sharp decrease of chronic (CG0/PG0) compared to acute conditions (CG1/PG1 and CG2/PG2) during the first and second COVID-19 waves of 2020, suggesting an increase of disease severity due to the pandemic. Nevertheless, since the increase in disease severity was limited compared to the reduction of ED admissions, it can be hypothesized that this latter finding has also partially been due to a decrease of inappropriate ED presentations.

More severe symptoms due to delayed access to hospital care have been reported in the pediatric population [4] and acute coronary syndrome cases [10,35]. Increased disease severity has also been described for abdominal conditions, with an increase of complicated cases among patients with diverticulitis [22,24,25] and acute appendicitis [23,36]. Cano-Valderaama [32] reported a statistically significant increase of time from symptom onset to ED presentation among patients with acute surgical conditions, leading to higher Sequential Organ Failure Assessment (SOFA) scores, lower laparoscopic interventions, higher morbidity, and longer lengths of stay, while Tartaglia et al. reported a higher percentage of patients requiring ICU admission during the pandemic [37].

Regarding the present study limitations, the main one is arguably represented by the lack of a further analysis of the ultimate impact on clinical outcomes of the observed results in terms of disease severity. This analysis would have been especially important considering that delayed surgical intervention has been associated with increased mortality in specific conditions like complicated diverticulitis [38]. Unfortunately, the allocation of patients from the ED to multiple surgical units after the surgical procedure made it impossible to collect reliable follow-up data and thus our analysis was restricted to the two main “junction points” during patients’ workflow (e.g., emergency department and pathology units). By tackling this open question, future studies would allow us to ascertain a potential additional health burden due to the COVID-19 pandemic. Moreover, by increasing the sample sizes of each analyzed condition, future multicentric studies would also allow multivariate analysis to assess the role of potential confounders.

## 5. Conclusions

To the best of the authors’ knowledge, this is the first integrated analysis of clinical, surgical, and pathological data proving the adverse consequences of COVID-19 on abdominal surgical emergencies. These findings support the importance of proactive measures to promptly address the whole spectrum of clinical and surgical urgencies, even during public health crises.

## Data Availability

The datasets used and/or analyzed during the current study are available from the corresponding author on reasonable request.

## Supplementary Materials

The following are available online at https://www.mdpi.com/article/10.3390/jcm10225254/s1, Figure S1: Total monthly ED accesses during the pre-pandemic years (2017–2019) and 2020, Figure S2: Trend of surgical activity for overall abdominal emergencies (A), cholecystitis (B), diverticulitis (C) and appendicitis (D) over time: comparison between the 2017–2019 period and 2020, Figure S3: Monthly distribution of patients undergoing surgery for appendicitis according to the clinical (A) and pathological (B) severity classification system: comparison between the 2017–2019 period and 2020, Table S1: Admissions to the ED in the analyzed months of 2020 compared to the pre-COVID-19 years (2017–2019).
