# Supplementary material for: The Adverse Impact of the COVID-19 Pandemic on Abdominal Emergencies: A Retrospective Clinico-Pathological Analysis
Source: J Clin Med. 2021 Nov 11;10(22):5254. doi: 10.3390/jcm10225254 (PMC8618829; doi:10.3390/jcm10225254)
Supplement: Supplementary file 1 [file jcm-10-05254-s001.zip › jcm-1426570-supplementary.pdf]

Journal of
Clinical Medicine

Supplementary Material: The Adverse Impact of the COVID-19 Pandemic on Abdominal Emergencies: A Retrospective Clinico-Pathological Analysis

Elena Vissio, Enrico Costantino Falco, Gitana Scozzari, Antonio Scarmozzino, Do An Andrea Trinh, Mario Morino, Mauro Papotti, Luca Bertero and Paola Cassoni

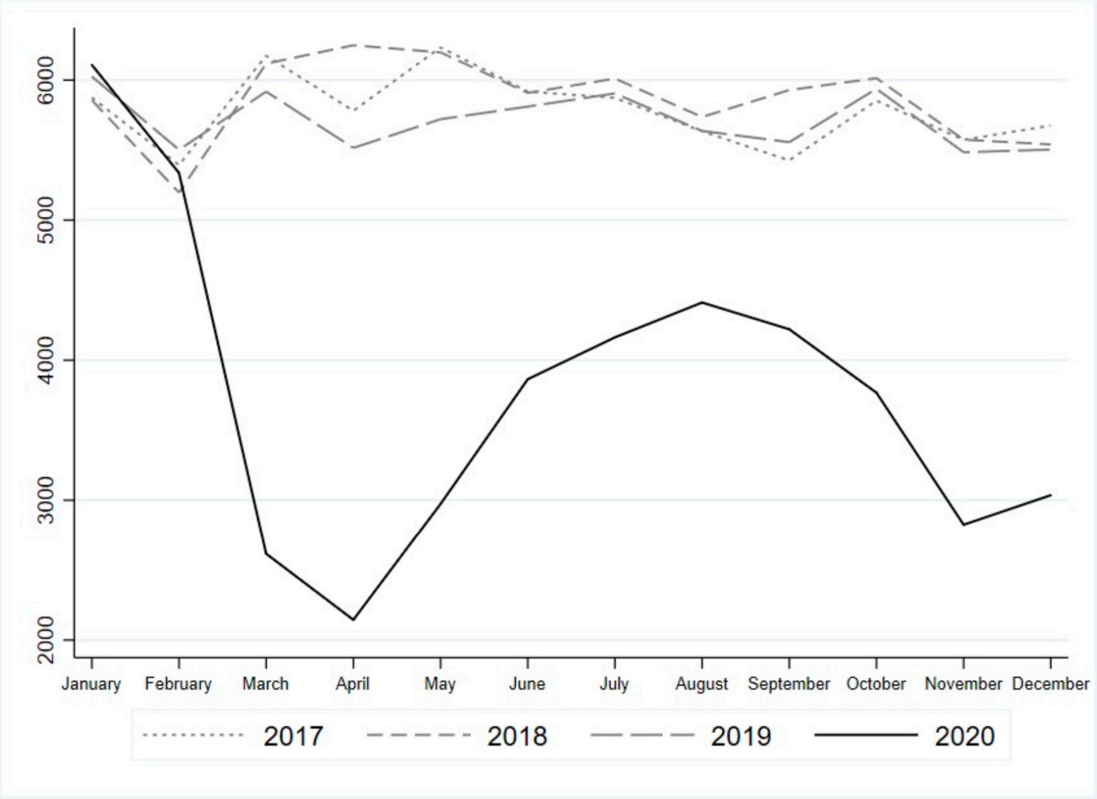

Figure S1. Total monthly ED accesses during the pre-pandemic years (2017–2019) and 2020.

Table S1. Admissions to the ED in the analyzed months of 2020 compared to the pre-COVID-19 years (2017–2019).

	DIVERTICULITIS		APPENDICITIS		CHOLECYSTITIS		TOTAL ABDOMINAL EMERGENCIES	
	Mean Value of 2017–2019 (Mean of Red and Yellow Codes)	2020 (Red and Yellow Codes)	Mean Value of 2017–2019 (Mean of Red and Yellow Codes)	2020 (Red and Yellow Codes)	Mean Value of 2017–2019 (Mean of Red and Yellow Codes)	2020 (Red and Yellow Codes)	Mean Value of 2017–2019 (Mean of Red and Yellow Codes)	2020 (Red and Yellow Codes)
**March**	22 (4)	9 (1)	21 (5)	12 (1)	56.0 (18.0)	25 (4)	99.0 (27.0)	46 (6)
**April**	22.7 (6)	10 (1)	16.3 (2.3)	10 (0)	58.0 (16.7)	24 (3)	97.0 (25.0)	44 (4)
**May**	26.7 (4)	13 (1)	22.7 (3.7)	23 (5)	61.0 (20.3)	35 (6)	110.3 (28.0)	71 (12)
**June**	22 (3.7)	13 (2)	19.3 (6.3)	16 (2)	52.7 (13.0)	46 (7)	94.0 (23.0)	75 (11)
**July**	30 (4.7)	17 (4)	16.3 (4.3)	21 (2)	49.3 (14.3)	31 (8)	95.7 (23.3)	69 (14)
**August**	20.7 (4.0)	16 (0)	11.0 (2.3)	22 (2)	51.0 (14.0)	38 (11)	82.7 (20.3)	76 (13)
**September**	32.7 (3.3)	16 (1)	21.0 (6.0)	13 (4)	55.7 (18.3)	56 (7)	109.3 (27.7)	85 (12)
**October**	26.7 (5.3)	14 (4)	20.0 (4.3)	11 (1)	67.7 (19.7)	36 (8)	114.3 (29.3)	61 (13)
**November**	36.3 (5.0)	10 (1)	17.3 (4.0)	13 (3)	58.7 (20.0)	40 (7)	112.3 (29.0)	63 (11)
**December**	22.7 (5.0)	17 (4)	13.3 (1.7)	16 (1)	46.0 (15.7)	25 (5)	82.0 (22.3)	58 (10)
**TOTAL**	262.3 (45.0)	135 (19)	178.3 (40.0)	157 (21)	556.0 (170)	356 (66)	996.7 (255.0)	648 (106)

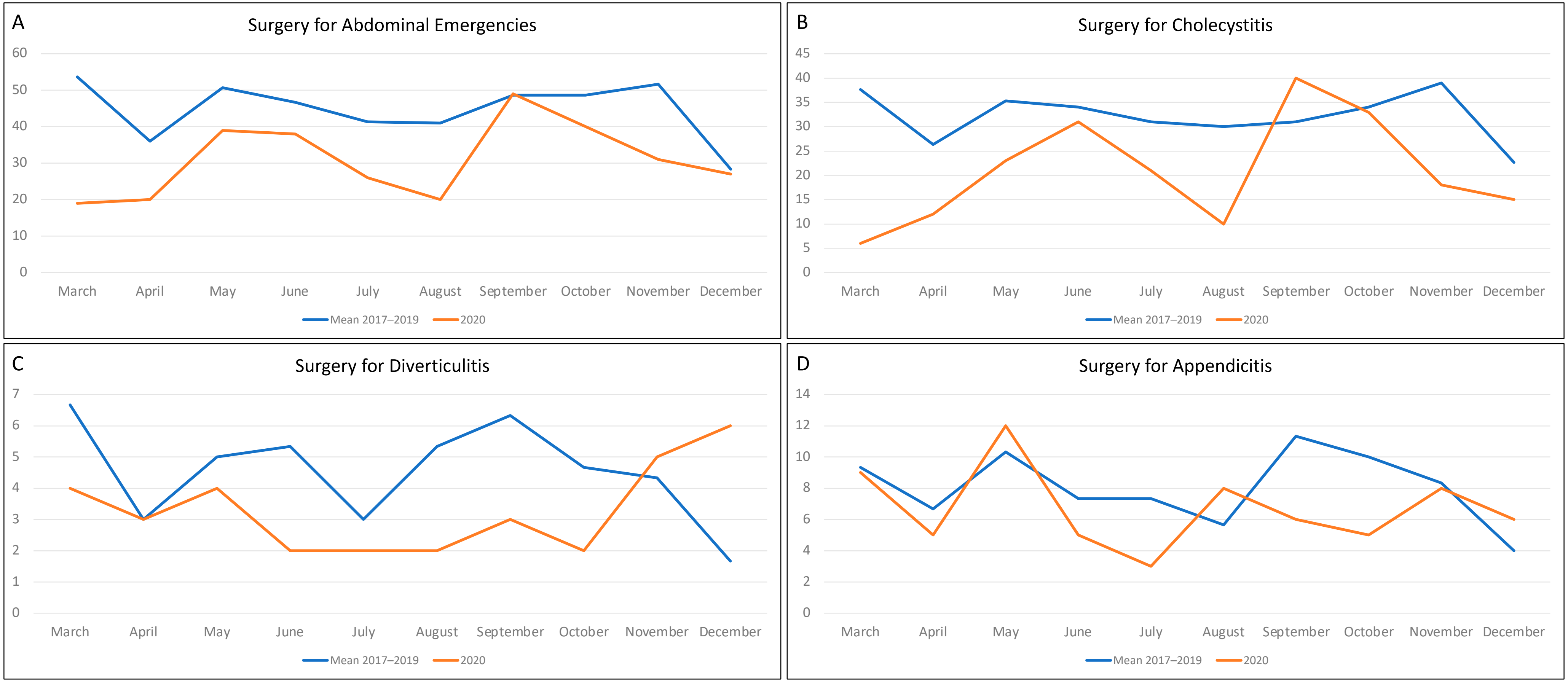

Figure S2. Trend of surgical activity for overall abdominal emergencies (**A**), cholecystitis (**B**), diverticulitis (**C**) and appendicitis (**D**) over time: comparison between the 2017–2019 period and 2020.

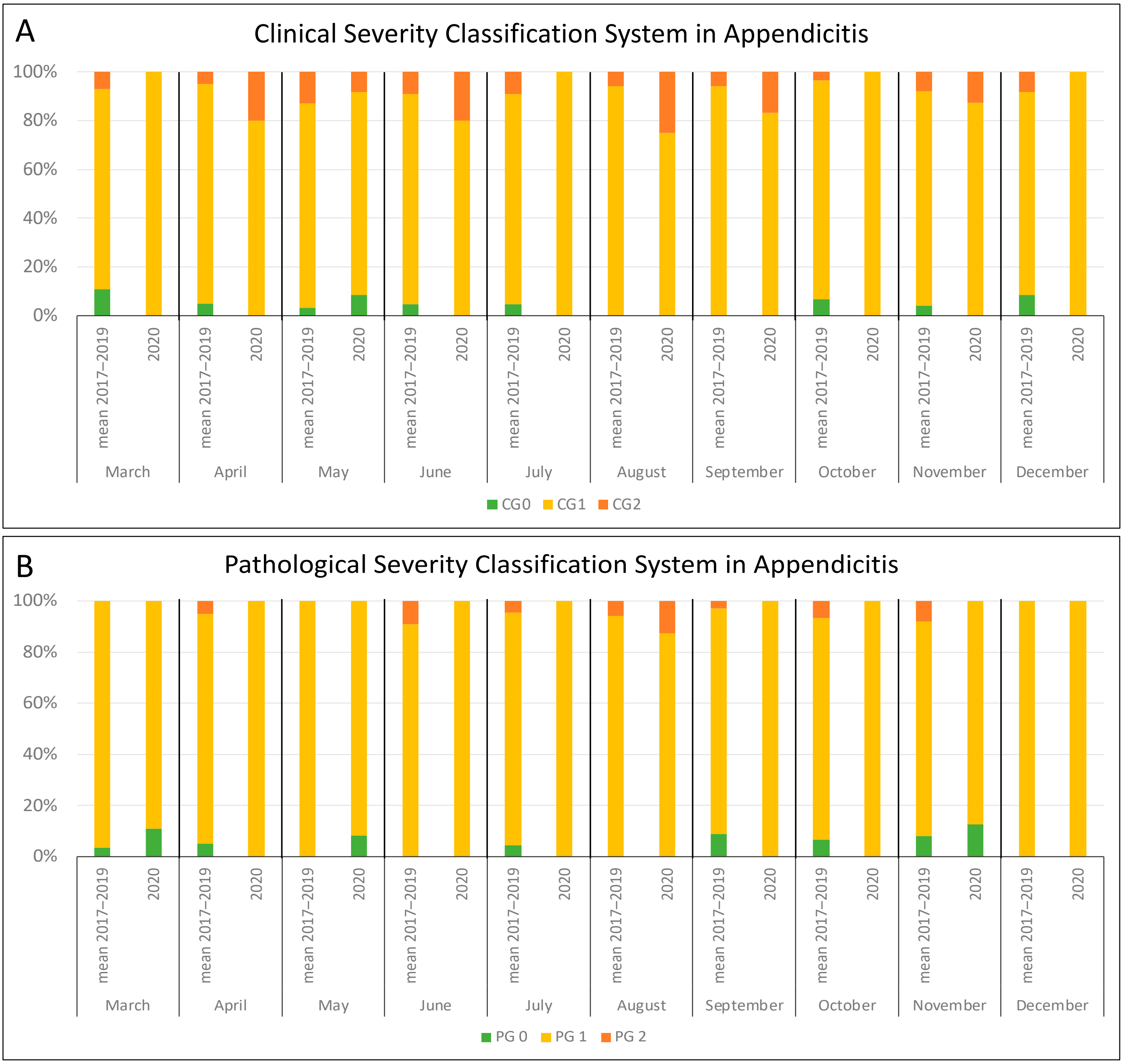

Figure S3. Monthly distribution of patients undergoing surgery for appendicitis according to the clinical (**A**) and pathological (**B**) severity classification system: comparison between the 2017–2019 period and 2020.
